# SPARC activates p38γ signaling to promote PFKFB3 protein stabilization and contributes to keloid fibroblast glycolysis

**DOI:** 10.1186/s41232-024-00357-y

**Published:** 2024-10-31

**Authors:** Yining Liu, Wei Zhang, Nan Lin, Zelei Yang, Yanxin Liu, Huaxia Chen

**Affiliations:** 1 Department of Burn and Plastic Surgery, the Affiliated Hospital of Qingdao University, Qingdao, 266100 Shandong People’s Republic of China; 2 Department of Burn and Plastic Surgery, Shandong Provincial Hospital Affiliated to Shandong First Medical University, No. 324 JingWu Road, Jinan, 250021 Shandong People’s Republic of China

**Keywords:** Keloid, Glycolysis, SPARC, P38γ, PFKFB3

## Abstract

**Background:**

Keloids are currently challenging to treat because they recur after resection which may affect patients’ quality of life. At present, no universal consensus on treatment regimen has been established. Thus, finding new molecular mechanisms underlying keloid formation is imminent. This study aimed to explore the function of secreted protein acidic and cysteine rich (SPARC) on keloids and its behind exact mechanisms.

**Methods:**

The expression of SPARC, p38γ, 6-phosphofructo-2-kinase/fructose-2,6-biphosphatase 3 (PFKFB3), α-SMA, and Ki67 in patients with keloid and bleomycin (BLM)-induced fibrosis mice was assessed utilizing western blot, qRT-PCR, and immunohistochemical staining. After transfected with pcDNA-SPARC, si-SPARC-1#, si-SPARC-2#, and si-p38γ, and treated with glycolytic inhibitor (2-DG) or p38 inhibitor (SB203580), CCK-8, EdU, transwell, and western blot were utilized for assessing the proliferation, migration, and collagen production of keloid fibroblasts (KFs).

**Results:**

SPARC, p38γ, and PFKFB3 were highly expressed in patients with keloid and BLM-induced fibrosis mice. SPARC promoted the proliferation, migration, and collagen production of KFs via inducing glycolysis. Moreover, SPARC could activate p38γ signaling to stabilize PFKFB3 protein expression in KFs. Next, we demonstrated that SPARC promoted the proliferation, migration, collagen production, and glycolysis of KFs via regulating p38γ signaling. In addition, in BLM-induced fibrosis mice, inhibition of p38γ and PFKFB3 relieved skin fibrosis.

**Conclusions:**

Our findings indicated that SPARC could activate p38γ pathway to stabilize the expression of PFKFB3, and thus promote the glycolysis of KFs and the progression of keloid.

**Supplementary Information:**

The online version contains supplementary material available at 10.1186/s41232-024-00357-y.

## Highlights

1. SPARC promotes KF glycolysis.

2. SPARC activates p38γ signaling to stabilize PFKFB3 expression.

3. SPARC promotes glycolysis of KFs via activating p38γ signaling

**Supplementary Information:**

The online version contains supplementary material available at 10.1186/s41232-024-00357-y.

## Background

Keloids are a growing and aggressive fibrotic lesion that most often arise at sites of cutaneous injury, and are caused by the overgrowth of granulation tissue or collagen during the healing process [1–3]. People of color have the highest prevalence, ranging from 4 to 16% [2, 4]. Keloids often present as the pain and pruritus in both lesional and perilesional except the apparent cosmetic problems [5]. Keloids are currently challenging to treat because they recur after resection which may affect patients’ quality of life [1]. At present, no universal consensus on treatment regimen has been established. Thus, finding new molecular mechanisms underlying keloid formation is imminent.

Secreted protein acidic and cysteine rich (SPARC) is a multifunctional glycoprotein belonging to the matricellular group of proteins [6]. SPARC is reported to participate in collagen deposition and has previously been proposed as a collagen companion [7]. In recent years, SPARC is discovered to modulate the progression of fibrotic diseases. Fan et al. [8] have suggested that SPARC knockdown attenuated TGF-β1-induced fibrotic functions via Smad2/3 pathways in human pterygium fibroblasts. Carvalheiro et al. [9] confirmed that SPARC is a critical pro-fibrotic mediator contributing to systemic sclerosis in a TGF-β dependent manner. The characteristic of keloid is the continuous accumulation of extracellular matrix (ECM) in connective tissue, primarily collagen [10]. Interestingly, our previous study has demonstrated that SPARC accelerated fibroblast proliferation, migration, and ECM synthesis in keloids through suppressing p53 pathway [11].

The effects and the behind exact mechanisms of SPARC on keloids may be complex and not fully understood. Therefore, we investigated the effect and potential mechanisms of SPARC on keloids. Our findings verified that SPARC could activate p38γ pathway to stabilize the expression of 6-phosphofructo-2-kinase/fructose-2,6-biphosphatase 3 (PFKFB3), and thus participate in the glycolysis of keloid fibroblasts and the progression of keloid.

## Methods

### Bioinformatic analysis

We used the Gene Set Enrichment Analysis (GSEA) software (http://software.broadinstitute.org/gsea/index.jsp) to analyze and enrich the SPARC-related pathways through using GSE182192 dataset [12]. In addition, the interaction of SPARC, p38γ, and PFKFB3 was plotted using GeneMANIA database.

### Patient samples

The experiments were approved by the Committees on the Ethics of Shandong Provincial Hospital Affiliated to Shandong First Medical University. Each participating individual wrote informed consents before enrolling in this study. Participants with keloid (*n* = 12) and healthy controls (*n* = 6) were recruited. Control samples of normal skin were taken from the perilesional skin after surgical removal of melanocytic nevus.

### Separation and culture of keloid fibroblasts

Separation of keloid fibroblasts (KFs) were separated based on our previous protocol [11]. Next, the isolated KFs were inoculated in the Dulbecco’s modified Eagle’s medium (Sigma-Aldrich, USA) containing 10% fetal bovine serum (FBS, R&D Systems, USA) and 1% antibiotics (Sigma-Aldrich, USA), and then cultured at 37 °C in a humidified 5% carbon dioxide atmosphere. Passage 3–5 KFs were used.

### Cell transfection and treatment

The empty vector and pcDNA-SPARC (OE-SPARC) were purchased from Genomeditech Biotechnology Co., Ltd. (Shanghai, China). si-SPARC-1# (5′-GGCCTGGATCTTCTTTCTCCT-3′), si-SPARC-2# (5′-GGAAGAAACTGTGGCAGAGGT-3′), si-p38γ (5′-GTGGCCATCAAGAAGCTGTAT-3′), and the Scramble control (5′-GTCCGTTTCGTCCCGTATCTT-3′) were purchased from Ribobio (Guangzhou, China). Transfection on KFs was carried out employing Lipofectamine® 3000 (Invitrogen, USA). After transfection of 48 h, their efficiencies were assessed employing western blot. Meanwhile, 5 mM glycolytic inhibitor (2-DG), 15 µM p38 inhibitor (SB203580) or 25 µg/mL protein synthesis inhibitor (CHX) was added to cells and incubated for 48 h, 12 h, or different times, respectively, at 37 °C with 5% carbon dioxide.

### CCK-8 assay

KFs (5 × 10^3^ cells/well) were inoculated into 96-well plates. After incubating at room temperature for 0, 1, 2, and 3 days, CCK-8 stock solution (20 µL, Sigma-Aldrich, USA) was added to each well, and then KFs were incubated at 37 °C for 2 h. In the end, the absorbance was determined by scanning at 450 nm with a microplate reader (Bio-Rad, USA).

### EdU assay

KF proliferation was tested employing an EdU cell proliferation kit (Beyotime, Shanghai, China). Shortly, the differently treated KFs were planted in 24-well plates (5 × 10^4^ cells/well) containing 50 µM EdU labeling solution and incubated for 3 h with gentle shaking. Next, DAPI reagent (Beyotime, Shanghai, China) was added to each well for cell nuclei staining. In the end, fluorescence detection was performed under a fluorescence microscope.

### Transwell assay

The differently treated KFs (8 × 10^4^ cells/well) were seeded into the upper Transwell chamber (8 µm) in a fresh serum-free medium. Besides, the lower chamber was filled with medium plus 10% FBS. After 24 h, the migrated cells were stained utilizing crystal violet (Solarbio, Beijing, China) for 10 min. Finally, migration cell numbers were counted under a fluorescence microscope.

### Glucose uptake and lactate production assay

The supernatant of KFs was collected at 48 h after seeding for assessing glucose and lactate concentrations utilizing the glucose uptake assay kit (Solarbio, China) and the lactate assay kit (Abcam, UK), respectively, following the manufacturers’ protocols.

### Seahorse assay

Glycolytic fluxes were evaluated by detecting extracellular acidification rate (ECAR) and oxygen consumption rate (OCR). In brief, the seahorse assays were carried out to quantitatively detect ECAR and OCR of KFs utilizing the Seahorse XF Glycolysis Stress Test Kit (Seahorse Bioscience, USA) and the Seahorse XF Cell Mito Stress Test Kit (Seahorse Bioscience, USA) in an XF24 extracellular analyzer (Seahorse Bioscience, USA), as per the manufacturer’s protocols.

### qRT-PCR

Total RNA from keloid, extra-lesional, or normal tissues was extracted employing TRIzol (Takara, Japan), and then reversed-transcribed into cDNA using a HiScript II 1st Strand cDNA Synthesis Kit (Vazyme, Nanjing, China) according to manufacturer’s instructions. Next, cDNA was amplified using a Taq Pro Universal SYBR qPCR Master Mix (Vazyme, Nanjing, China) on the LightCycler 480 real-time PCR system (Roche Applied Science, USA). The primer sequences for qRT-PCR of genes are provided by Tsingke (Beijing, China) as follows: SPARC, 5′-CCCGCTTTTTCGAGACCTGT-3′ (forward), 5′-TCCTTGTCGATATCCTTCTGCTT-3′ (reverse) and β-actin, 5′- ACAGAGCCTCGCCTTTGCC-3′ (forward), 5′- GATATCATCATCCATGGTGAGCTGG-3′ (reverse).

### Western blot analysis

Radioimmunoprecipitation assay buffer (Beyotime, Shanghai, China) was used to extract proteins from cells and tissues. Next, the protein concentration was quantified employing a BCA assay kit (Beyotime, Shanghai, China). Afterwards, the protein was run on a SDS-PAGE and blotted onto a PVDF membrane. After blocking with 5% nonfat milk, the membranes were incubated with the primary antibodies in 5% milk-TBST overnight at 4 °C using the following concentrations: SPARC (1:500, no. ab290636 and ab225716, Abcam, UK), p38γ (1:1000, no. 20184–1-AP, Proteintech, USA), PFKFB3 (1:1000, no. 13763–1-AP, Proteintech, USA), α-SMA (1:1000, no. #19245, Cell Signaling Technology, USA), Fibronectin (1:500, no. ab2413, Abcam, UK), Collagen I (1:500, no. ab138492, Abcam, UK), Collagen III (1:500, no. ab184993, Abcam, UK) and β-actin (1:2000, no. ab8227, Abcam, UK). Next, the goat anti-rabbit IgG H&L (HRP) secondary antibody (1:5000, ab6721, Abcam, UK) was incubated with the membranes for 1 h prior to detection with ECL detection reagent (Beyotime, Shanghai, China).

### Immunohistochemistry staining

Paraffin Sections (4-µm-thick) of human and mice were dewaxed, rehydrated, and washed in deionized water as per the standard protocol. After that, the sections were treated with 3% hydrogen peroxide for repressing endogenous peroxidase activity and incubated in boiling citrate buffer for antigen retrieval. Following blocking with 5% goat serum, the sections were incubated with primary antibodies (SPARC-mouse, 1:100, no. ab290636, Abcam, UK; SPARC-human, 1:100, no. ab225716, Abcam, UK; p38γ, 1:50, no. 20184–1-AP, Proteintech, USA; PFKFB3, 1:100, no. 13763–1-AP, Proteintech, USA; α-SMA, 1:50, no. #19245, Cell Signaling Technology, USA; Ki67, 1:100, no. ab15580, Abcam, UK) at 4 °C overnight. Then, sections were further incubated with goat anti-rabbit immunoglobulin for 1 h at room temperature. After rinsing, the sections were developed with diaminobenzidine tetrahydrochloride, counter-stained utilizing hematoxylin, and then observed under a fluorescence microscope (Olympus, Japan).

### Co-immunoprecipitation (co-IP)

KFs were lysed with lysis buffers containing 1% N-P40, 20 mmol/L Tris–HCL (pH 7.4), 200 mmol/L NaCl, and complete protease inhibitor mixture, and incubated on ice for 1 h. For exogenous IP, the filtered supernatant was subjected to IP with anti-FLAG M2 affinity resin (Sigma-Aldrich, USA) at 4 °C overnight. For endogenous IP, the filtered supernatant was incubated with anti-PFKFB3, anti-p38γ, and anti-IgG overnight at 4 °C. Next, protein A/G agarose beads (MedChemExpress, USA) were added and incubated on a rotary mixer at 4 °C for 4 h, followed by four times with lysis buffer and one time with PBS. In the end, the immunoprecipitated protein was eluted and then subjected to immunoblotting.

### Fibrosis animal models

Healthy female C57BL/6 mice (6–8 weeks, Pengyue, Jinan, China) were used for a bleomycin (BLM)-induced skin fibrosis model [13]. Shortly, 100 µl of BLM (2 U/ml) or normal saline was subcutaneously injected to a single location on the shaved back once daily for a total of 28 days. Next, the injected skin was cut. Moreover, to inhibit p38 and PFKFB3 proteins, the mice were administered either SB203580 (40 mg/kg, intraperitoneal) or 3-(3-pyridinyl)-1-(4-pyridinyl)-2-propen-1-one (3PO, 35 mg/kg, intraperitoneal). Animal experiments were approved by the Committees on the Ethics of Shandong Provincial Hospital Affiliated to Shandong First Medical University.

### Histological analysis of skin

After euthanization, skin sections were fixed in 10% formalin, dehydrated, embedded in paraffin, and cut into 4-µm-thick sections. After deparaffinizing utilizing xylene, rehydrating utilizing successive immersion in descending concentrations of alcohol, and washing in deionized water, the sections were stained with hematoxylin and eosin (H&E, Beyotime, Shanghai, China) and Masson’s trichrome (Sigma-Aldrich, USA) according to routine procedures. Images were photographed with a fluorescence microscope (Olympus, Japan) and analyzed via utilizing Image-Pro Plus Version 6.0.

### Statistical analysis

SPSS 22.0 and GraphPad Prism 8.0 were employed for statistical analysis. Data are shown as mean ± standard error of the mean. Differences between groups were compared by Student’s *t*-test or analysis of variance (ANOVA) test. *p* < 0.05 was considered statistically significant.

## Results

### SPARC was highly expressed in human keloids

**Fig. 1:**
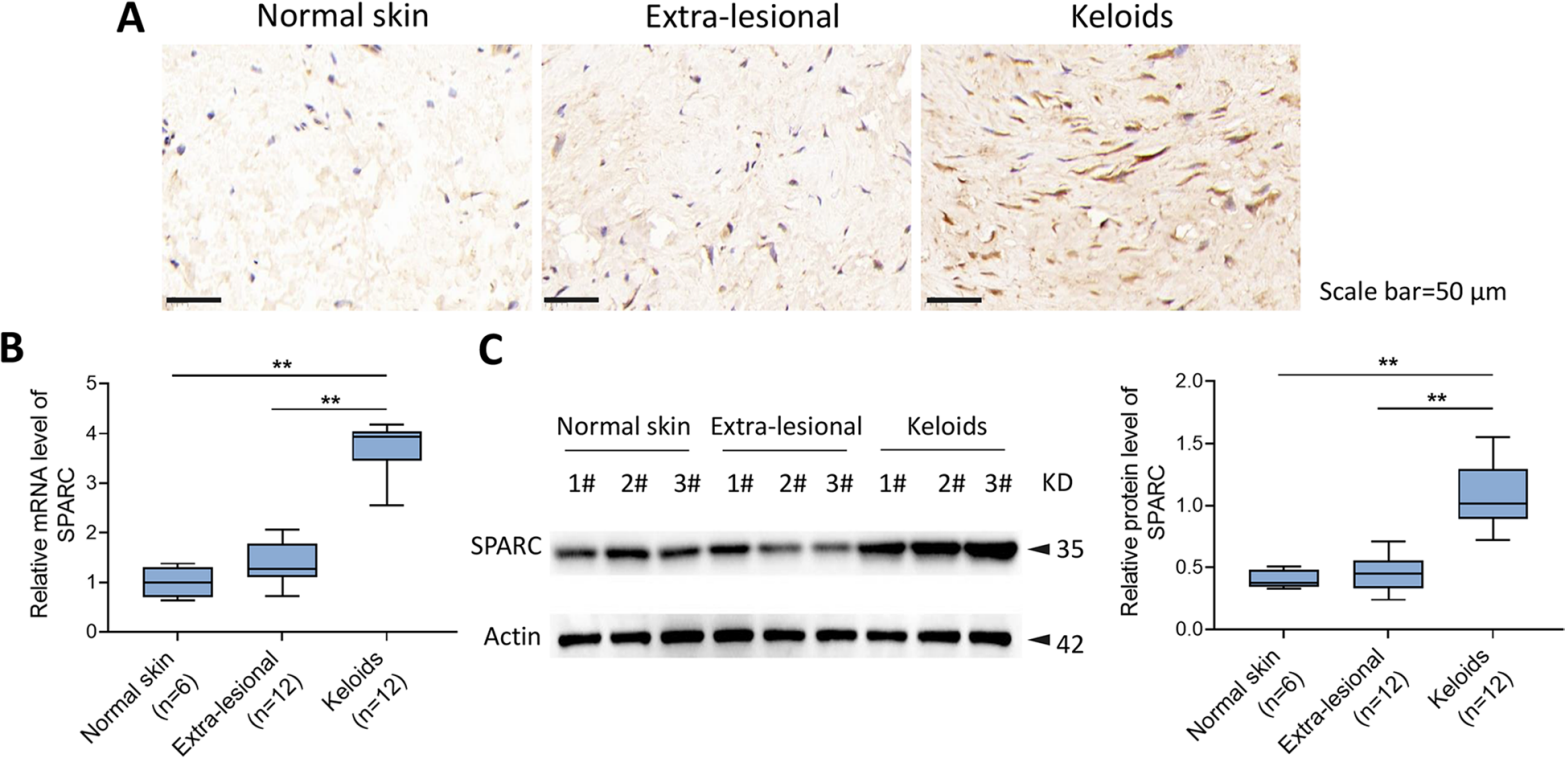
SPARC was highly expressed in human keloids. Immunohistochemical staining for SPARC (**A**); qRT-PCR (**B**) and western blot (**C**) for evaluating SPARC level. ^**^P < 0.01

The most striking feature that distinguishes normal skin from human keloid samples is the presence of undulating dermal junctions and skin appendages in the dermis, which thickens the keloid epidermis [14]. We detected the expression of SPARC in keloid, extra-lesional, and normal skin tissues through employing immunohistochemistry staining, qRT-PCR, and western blot. These results manifested that SPARC was significantly high-expressed in keloid tissues, when compared with normal skin tissues and extra-lesional tissues of patients with keloid (Fig. 1A–C).

### SPARC promoted the proliferation, migration, and collagen production in KFs

**Fig. 2:**
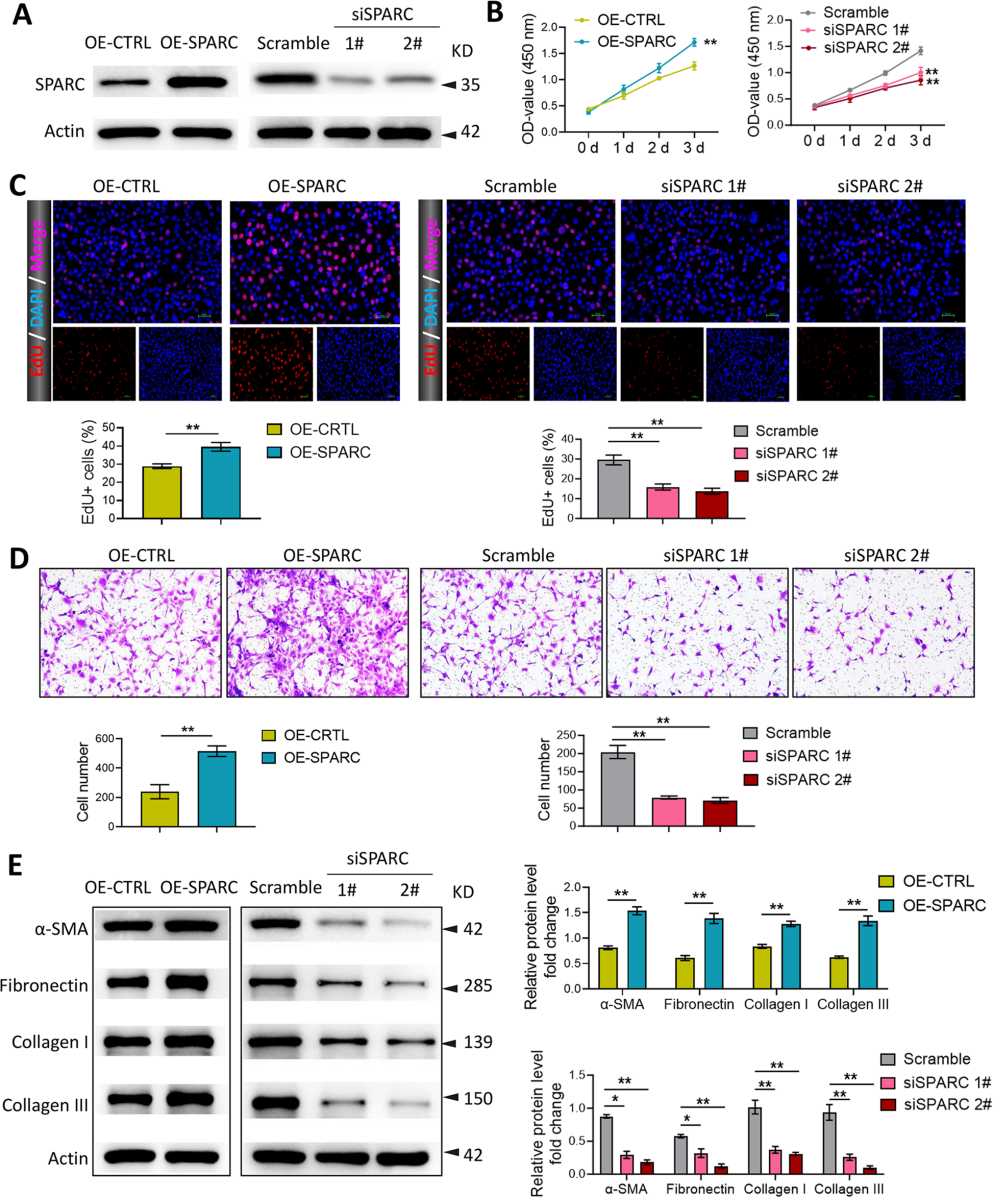
SPARC promoted the proliferation, migration, and collagen production in KFs. KFs were transfected with pcDNA-SPARC, si-SPARC-1# and si-SPARC-2#. **A** Western blot was applied for evaluating SPARC level; **B** CCK-8 and **C** EdU assays were utilized for assessing KF proliferation; **D** transwell was employed for detecting KF migration; **E** western blot was applied for evaluating α-SMA, Fibronectin, Collagen I, and Collagen III expression. ^*^P < 0.05, ^**^P < 0.01

After transfection with OE-SPARC, si-SPARC-1#, and si-SPARC-2#, the transfection efficiency was detected using western blot (Fig. 2A). Moreover, the data of CCK-8 and EdU assays manifested that the proliferation of KFs was promoted by the upregulation of SPARC, but suppressed by the downregulation of SPARC (Fig. 2B and C). Similarly, we also discovered that SPARC overexpression significantly elevated the migration of KFs, while SPARC knockdown notably reduced the migration (Fig. 2D). In addition, western blot results demonstrated that the expression of α-SMA, Fibronectin, Collagen I, and Collagen III was markedly upregulated in KFs after SPARC overexpression, but notably downregulated after SPARC knockdown (Fig. 2E). To sum up, SPARC could promote the proliferation, migration, and collagen production in KFs.

### SPARC promoted the proliferation, migration, and collagen production of KFs via inducing glycolysis

**Fig. 3:**
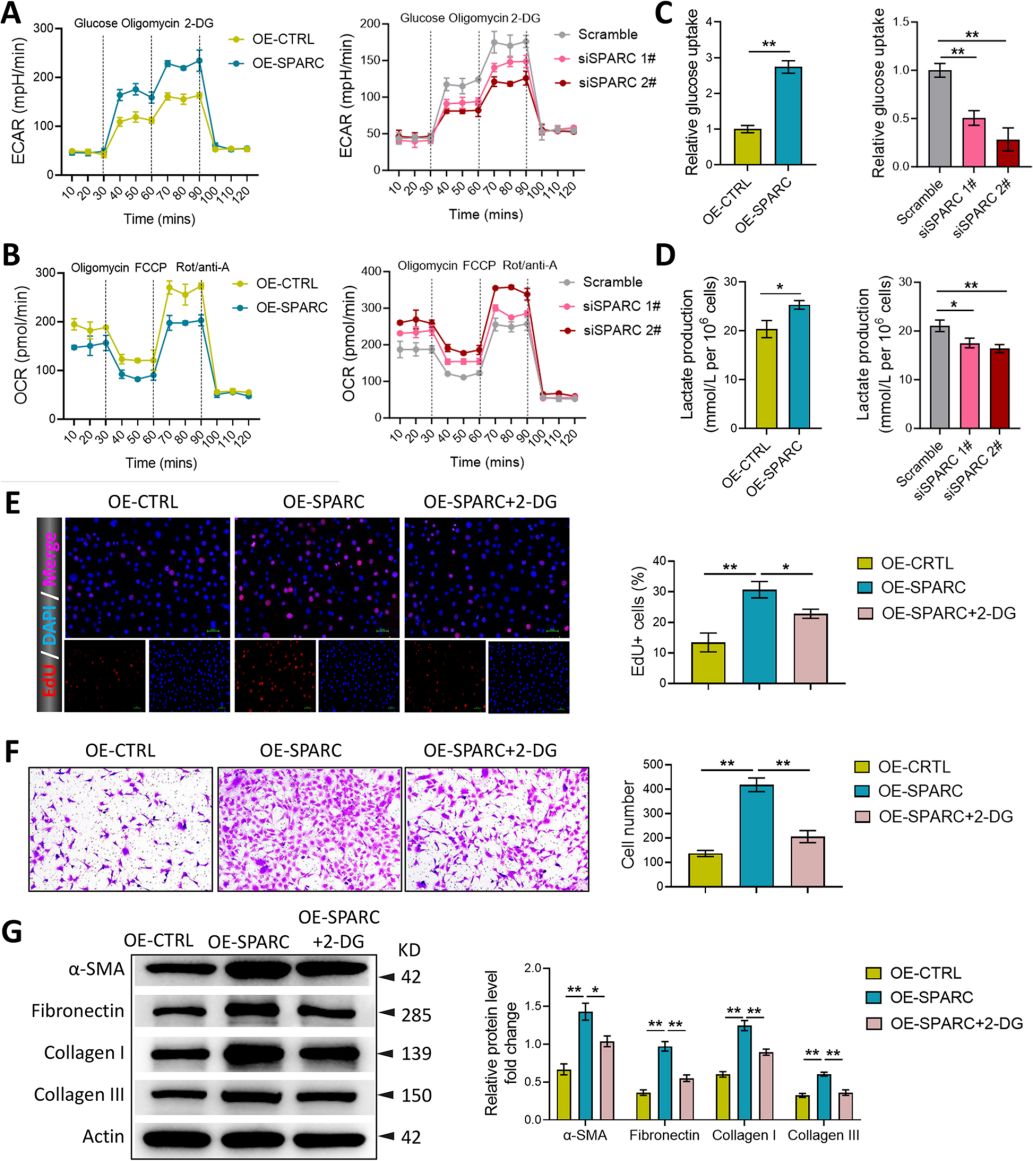
SPARC promoted the proliferation, migration, and collagen production of KFs via inducing glycolysis. After transfected with pcDNA-SPARC, si-SPARC-1#, and si-SPARC-2#, the effects of SPARC overexpression or silencing on ECAR (**A**), OCR (**B**), glucose uptake (**C**), and lactate production (**D**) were investigated in KFs. After transfected with pcDNA-SPARC and treated with 2-DG, proliferation was assessed employing EdU assay (**E**); migration was evaluated utilizing transwell assay (**F**); α-SMA, Fibronectin, Collagen I, and Collagen III expression was measured via western blot (**G**). ^*^P < 0.05, ^**^P < 0.01

Firstly, we tested ECAR and OCR using a XF24 extracellular analyzer to measure the effects of SPARC on glycolytic fluxes. It was discovered that the upregulation of SPARC significantly accelerated ECAR and repressed OCR (Fig. 3A and B). On the contrary, the downregulation of SPARC markedly inhibited ECAR and promoted OCR (Fig. 3A and B). Subsequently, we demonstrated that SPARC overexpression notably increased glucose uptake and lactate production in KFs, while SPARC silencing dramatically decreased glucose uptake and lactate production (Fig. 3C and D). Next, to investigate the effect of glycolysis on SPARC-regulated cell proliferation, migration, and collagen production, the glycolysis inhibitor (2-DG) was added into the cell culture medium. As shown in Fig. 3E and F, 2-DG significantly inhibited the promoting effects of SPARC overexpression on cell proliferation and migration. Moreover, the increased expression of α-SMA, Fibronectin, Collagen I, and Collagen III induced by SPARC overexpression in KFs was partially abolished by 2-DG treatment (Fig. 3G). These above observations suggested that SPARC could promote the proliferation, migration, and collagen production of KFs via inducing glycolysis.

### SPARC activated p38γ signaling to stabilize PFKFB3 protein expression

**Fig. 4:**
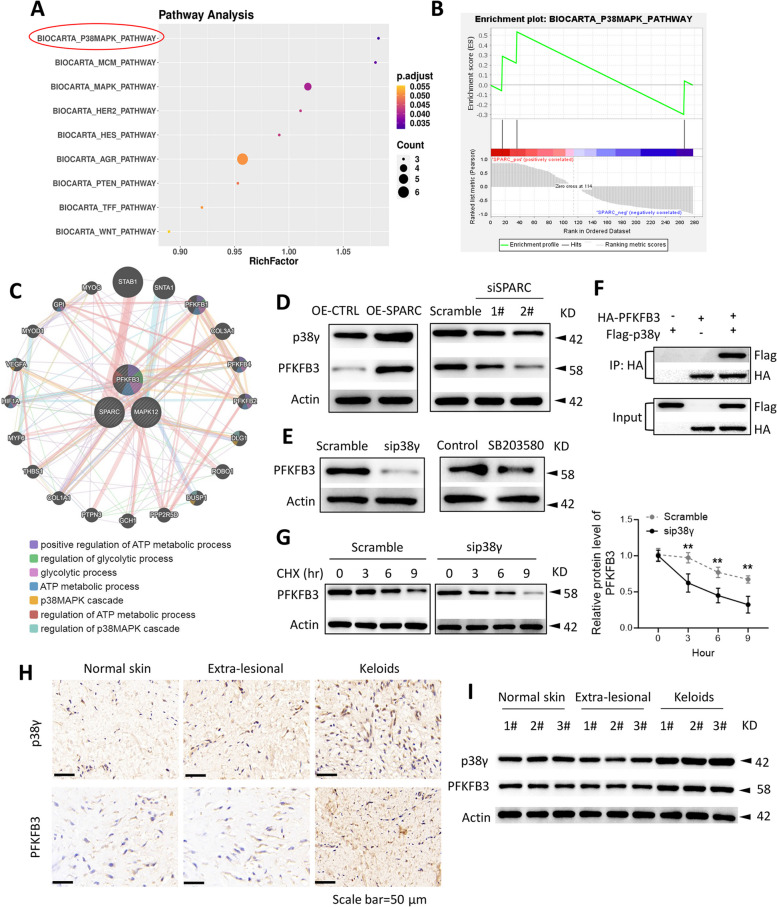
SPARC activated p38γ signaling to stabilize PFKFB3 protein expression. **A**, **B** GSEA manifested that SPARC regulated p38 pathway. **C** The interaction between SPARC, p38γ, and PFKFB3 in PPI network. **D** After transfected with pcDNA-SPARC, si-SPARC-1#, and si-SPARC-2#, p38γ and PFKFB3 expression was measured via western blot. **E** After transfected with si-p38γ or treated with SB203580, PFKFB3 expression was measured via western blot. **F** Western blot of co-IP was performed to verify the binding ability between p38γ and PFKFB3 in KFs. **G** After transfected with si-p38γ and treated with CHX, p38γ and PFKFB3 expression in KFs was measured via western blot. **H** Immunohistochemical staining and **I** western blotting for p38γ and PFKFB3 in human keloids and extra-lesional samples. ^**^P < 0.01

GSEA was used to enrich the pathways modulated by SPARC, and results showed that SPARC positively regulated the p38MAPK pathway (Fig. 4A and B). Then, through mapping the PPI network, we found the interaction between SPARC, p38γ (MAPK12), and PFKFB3 (a key glycolytic enzyme) (Fig. 4C). Interestingly, p38γ can participate in the glycolysis of tumor cells by stabilizing the expression of PFKFB3 protein [15]. Thus, we hypothesized that SPARC might regulate the p38γ pathway to stabilize the expression of PFKFB3 protein, thereby participating in the glycolysis of KFs and keloid progression. The results of the western blot showed that the expression of p38γ and PFKFB3 was increased after SPARC overexpression, but decreased after SPARC silencing (Fig. 4D). Besides, the silence of p38γ or the treatment of SB203580 significantly repressed the expression of PFKFB3 in KFs (Fig. 4E). Next, co-IP assay results confirmed the association of p38γ and PFKFB3 (Fig. 4F). As shown in Fig. 4G, p38γ silencing significantly inhibited the stability of PFKFB3 protein. The results of Fig. 4H and I showed that p38γ and PFKFB3 were overexpressed in keloid tissues, when compared with normal skin tissues and extra-lesional tissues of patients with keloid. These results confirm our hypothesis that SPARC could stabilize the expression of PFKFB3 by regulating the p38γ pathway.

### SPARC promotes the proliferation, migration, collagen production, and glycolysis of KFs via regulating p38γ signaling

**Fig. 5:**
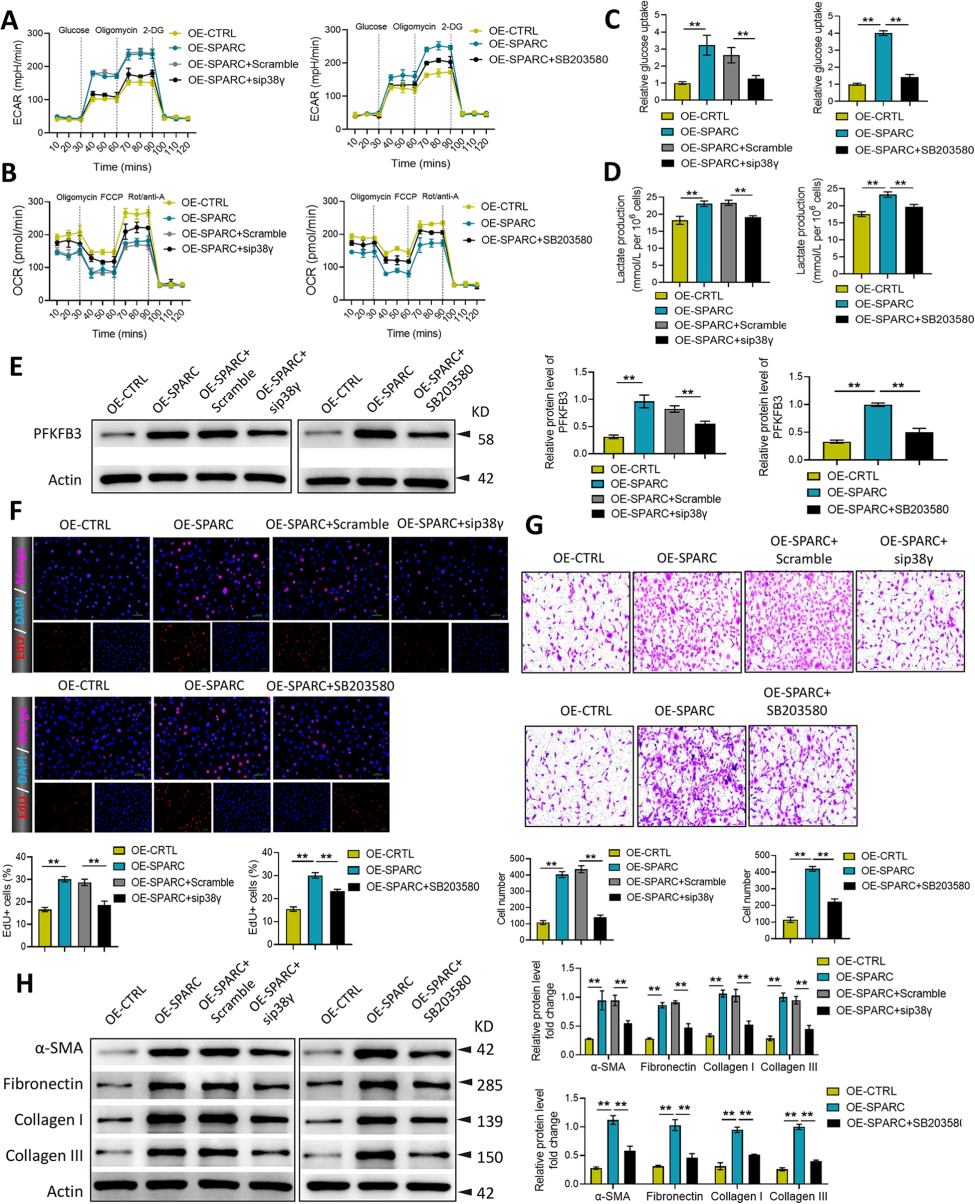
SPARC promotes the proliferation, migration, collagen production, and glycolysis of KFs via regulating p38γ signaling. After transfected with pcDNA-SPARC and si-p38γ, and treated with SB203580, ECAR (**A**), OCR (**B**), glucose uptake (**C**), and lactate production (**D**) were investigated in KFs. PFKFB3 expression was measured via western blot (**E**). The proliferation and migration were assessed using EdU (**F**) and transwell assay (**G**). α-SMA, Fibronectin, Collagen I, and Collagen III expression was measured via western blot (**H**). ^*^P < 0.05, ^**^P < 0.01

As seen in Fig. 5A and B, the silence of p38γ and SB203580 treatment significantly reversed the promoting effect of SPARC overexpression on ECAR and the inhibiting effect on OCR. Moreover, the silence of p38γ and SB203580 treatment significantly alleviated the promoting effect of SPARC overexpression on glucose uptake and lactate production in KFs (Fig. 5C and D). Then, the silence of p38γ and SB203580 treatment significantly inhibited the upregulation of PFKFB3 by SPARC overexpression (Fig. 5E). The data of EdU and transwell assays demonstrated that the elevated proliferation and migration of KFs caused by SPARC overexpression markedly abolished after p38γ silencing or SB203580 treatment (Fig. 5F and G). Furthermore, the increased expression of α-SMA, Fibronectin, Collagen I, and Collagen III induced by SPARC overexpression in KFs was reversed after p38γ silencing or SB203580 treatment (Fig. 5H).

### SPARC, p38γ, and PFKFB3 were increased in the skin of BLM-induced fibrosis model in C57BL/6 mice

**Fig. 6:**
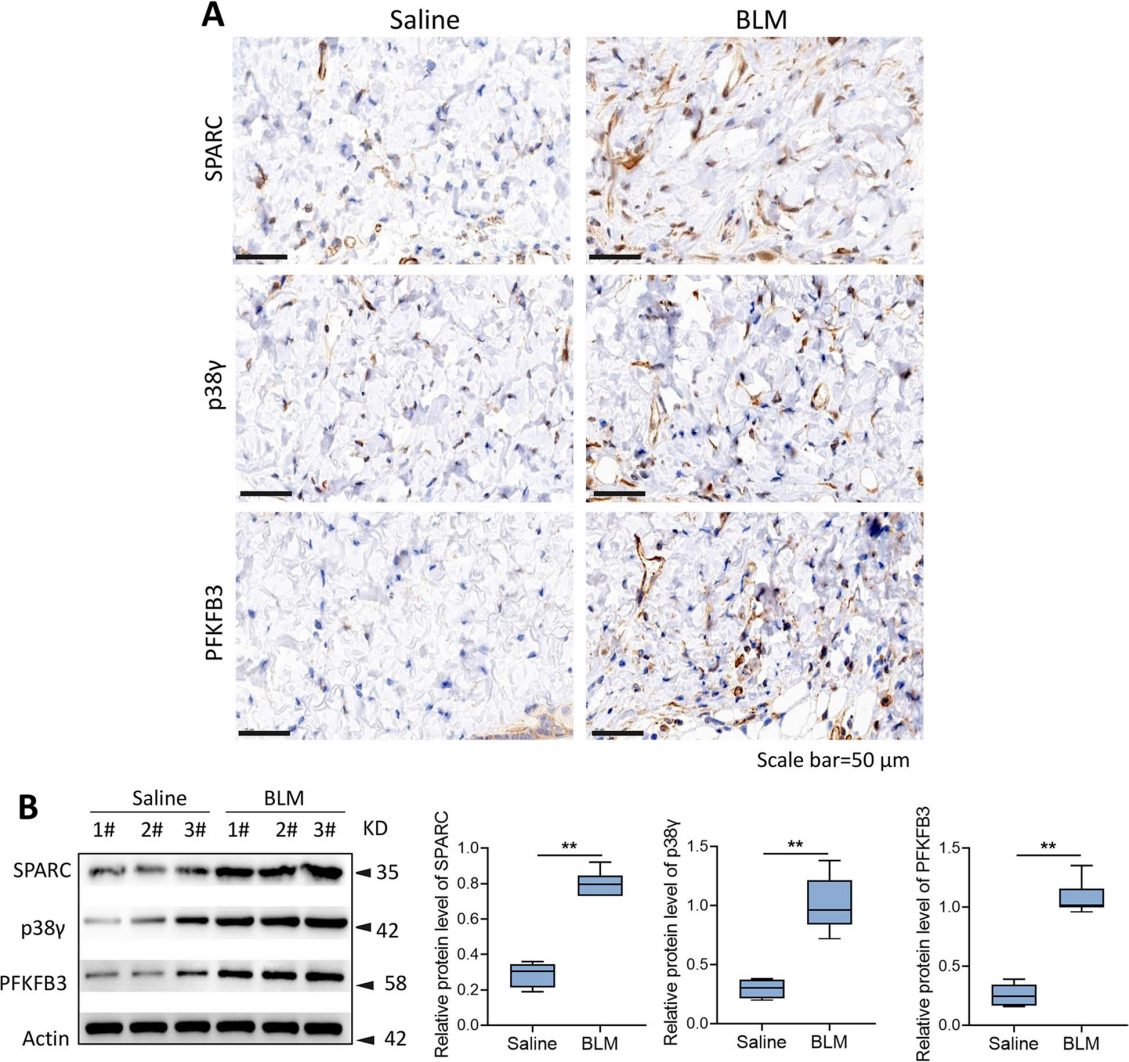
SPARC, p38γ, and PFKFB3 were increased in the skin of BLM-induced fibrosis mouse model. **A** Immunohistochemical staining for SPARC, p38γ, and PFKFB3. **B** Western blot for assessing SPARC, p38γ, and PFKFB3 expression. ^**^P < 0.01

The BLM-induced fibrosis mouse model has been widely used to investigate the mechanisms of skin fibrosis and to test potential therapeutic approaches [16]. In this study, a BLM-induced fibrosis mouse model was used to further confirm the regulating roles of SPARC in vivo. As Fig. 6A showed, when compared with the saline group, the expression of SPARC, p38γ, and PFKFB3 in the skin tissues of the BLM group was elevated. Similarly, the upregulated expression of SPARC, p38γ, and PFKFB3 was also confirmed using western blot in the skin tissues of the BLM group (Fig. 6B). Collectively, these data indicated that SPARC, p38γ, and PFKFB3 were upregulated in the skin of BLM-induced fibrosis model in C57BL/6 mice.

### Inhibition of p38γ or PFKFB3 relieved BLM-induced skin fibrosis in vivo

**Fig. 7:**
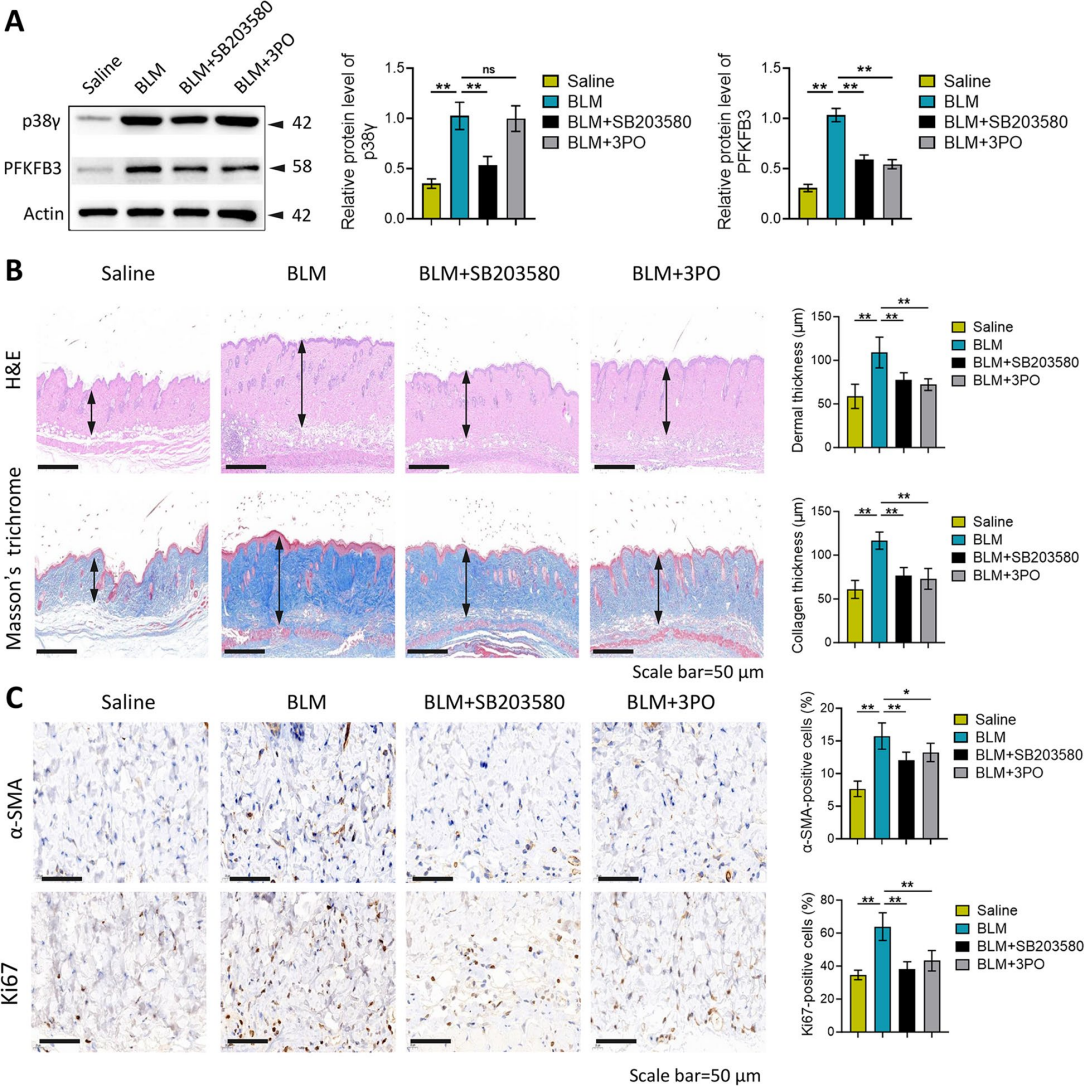
Inhibition of p38γ and PFKFB3 relieved BLM-induced skin fibrosis in vivo. After injected with SB203580 and 3PO, p38γ and PFKFB3 expression in BLM-induced mice skin tissues was measured employing western blot (**A**). The dermal thickness and collagen thickness of mice skin tissues were detected utilizing H&E and Masson’s trichrome staining (**B**). Immunohistochemical staining for α-SMA and Ki67 in BLM-induced mice skin tissues (**C**). ^*^P < 0.05, ^**^P < 0.01

Western blot manifested that intraperitoneal injection of SB203580 significantly reversed the overexpression of p38γ and PFKFB3 in mice skin induced by BLM (Fig. 7A). Meanwhile, injection of 3PO had no effect on the overexpression of p38γ induced by BLM, but inhibited the overexpression of PFKFB3 (Fig. 7A). As shown in Fig. 7B, compared with the saline group, the dermis and collagen in the skin tissues of the BLM group were significantly thickened. Intraperitoneal injection of SB203580 or 3PO could significantly inhibit dermal and collagen thickening (Fig. 7B). Besides, the skin tissues of BLM group mice had more α-SMA and Ki67 positive cells, when compared with the saline group (Fig. 7C). Intraperitoneal injection of SB203580 or 3PO significantly decreased α-SMA and Ki67 positive cells (Fig. 7C). All these results implied that inhibition of p38γ and PFKFB3 could relieve BLM-induced skin fibrosis in vivo.

## Discussion

In this study, we demonstrated that SPARC was upregulated in human keloid tissues and in skin tissues of BLM-induced fibrosis mice. The proliferation, migration, and collagen production of KFs were promoted by the upregulation of SPARC, but repressed by the downregulation of SPARC, which was consistent with our previous findings [11]. However, the precise molecular mechanism of SPARC on keloids is complex and not fully understood at present.

In recent years, more and more studies have confirmed that metabolic disturbances can occur in keloids, and glycolysis performs a vital role in keloid pathogenesis [17–19]. Huang et al. [20] have reported that PTB may regulate aerobic glycolysis and the cell functions of KFs through alternative splicing of PKM. Our findings showed that the upregulation of SPARC accelerated ECAR and repressed OCR. The effect of SPARC silencing on ECAR and OCR was the opposite. Besides, SPARC overexpression increased glucose uptake and lactate production in KFs, while SPARC silencing decreased them. Together, we believed that SPARC could promote glycolysis in KFs. Inhibition of glycolysis can reduce the proliferation, migration, and collagen I expression in KFs [21]. Wang et al. [22] have proved that PI3K/AKT pathway could promote proliferation and inhibit apoptosis in KFs under hypoxia by regulating glycolysis. Li et al. [23] have reported that the inhibition of glycolysis with 2-DG could inhibit the proliferation of KFs in a dose-dependent and time-dependent manner. In this study, 2-DG treatment significantly decreased proliferation, migration, and collagen production induced by SPARC overexpression in KFs, implying that SPARC could promote the proliferation, migration, and collagen production of KFs via inducing glycolysis.

A major driver of glycolysis is PFKFB3, which can generate fructose-2,6-bisphosphate (F2,6BP), the most influential allosteric activator of the glycolytic rate-limiting enzyme phosphofructokinase-1 (PFK1) [24–27]. Mejias et al. [28] have suggested that PFKFB3 overexpression can cause glycolysis and activate hepatic stellate cells, thus accelerating liver fibrosis. Chen et al. [29] have proved that inhibiting PFKFB3-driven glycolysis in myofibroblasts abolishes pulmonary fibrosis. The study by Wang et al. has indicated that p38γ can participate in the glycolysis process of tumor cells by stabilizing the expression of PFKFB3 protein [15]. Collectively, we speculated that SPARC might regulate the p38γ pathway to stabilize the expression of PFKFB3, and thus participated in the glycolysis process of KFs and the progression of keloid. Through the analysis of bioinformatics, SPARC positively regulates the p38MAPK pathway, and the interaction between SPARC, p38γ, and PFKFB3 is discovered. Next, we demonstrated that SPARC activated p38γ signaling to stabilize PFKFB3 protein expression through a series of experiments. p38γ, a stress-activated member of the MAPK family, has a common and specific role in signal transduction compared to other p38 proteins [30]. A study by Li et al. has proved that the high expression of osteomodulin can accelerate the hyperproliferation of KFs, migration, and excess ECM synthesis through activating the p38MAPK pathway [31]. Zhang et al. [32] report that 2-Methoxyestradiol could inhibit the proliferative ability in KFs through the p38MAPK pathway. Our results demonstrated that both p38γ silencing and SB203580 treatment could reverse the increased proliferation, migration, collagen production, and glycolysis induced by SPARC overexpression in KFs, suggesting that SPARC could promote the proliferation, migration, collagen production, and glycolysis of KFs via regulating p38γ signaling. In addition, the upregulation of SPARC, p38γ, and PFKFB3 were discovered in the skin of BLM-induced fibrosis mice model, and the inhibition of p38γ and PFKFB3 could relieve BLM-induced skin fibrosis.

## Conclusion

In conclusion, our work demonstrated that SPARC could activate the p38γ pathway to stabilize the expression of PFKFB3, and thus promote the glycolysis of KFs and the progression of keloid, implying that SPARC/p38γ/PFKFB3 signaling axis may be a new molecular mechanism in the therapy of keloids.

## Supplementary Information

Supplementary Material 1.

## Data Availability

The datasets used and/or analyzed during the current study are available from the corresponding author on reasonable request.
