# Supplementary material for: SPARC activates p38γ signaling to promote PFKFB3 protein stabilization and contributes to keloid fibroblast glycolysis
Source: Inflamm Regen. 2024 Oct 31;44:44. doi: 10.1186/s41232-024-00357-y (PMC11529245; doi:10.1186/s41232-024-00357-y)
Supplement: Supplementary file 1 — Supplementary Material 1. [file 41232_2024_357_MOESM1_ESM.docx]

Figure 1C

SPARC







Actin







Figure 2A-1

SPARC







Actin







Figure 2A-2

SPARC







Actin







Figure 2E-1

α-SMA







Fibronectin







Collagen I







Collagen II







Actin







Figure 2E-2

α-SMA







Fibronectin







Collagen I







Collagen II







Actin







Figure 3G

α-SMA

**



**

Fibronectin







Collagen I







Collagen II







Actin







Figure 4D-1

p38γ







PFKFB3







Actin







Figure 4D-2

p38γ







PFKFB3







Actin







Figure 4E-1

PFKFB3







Actin







Figure 4E-2

PFKFB3







Actin







Figure 4F

IP: Flag







IF: HA







Input: Flag







Input: HA







Figure 4G-1

PFKFB3







Actin







Figure 4G-2

PFKFB3







Actin







Figure 4I

p38γ







PFKFB3







Actin







Figure 5E-1

PFKFB3







Actin







Figure 5E-2

PFKFB3







Actin







Figure 5H-1

α-SMA







Fibronectin







Collagen I







Collagen II







Actin

Figure 5H-2

α-SMA

Fibronectin

Collagen I

Collagen II

Actin

Figure 6B

SPARC

p38γ

PFKFB3

Actin

Figure 7A

p38γ

PFKFB3

Actin
